# Zoonotic helminths affecting the human eye

**DOI:** 10.1186/1756-3305-4-41

**Published:** 2011-03-23

**Authors:** Domenico Otranto, Mark L Eberhard

**Affiliations:** 1 Dipartimento di Sanità Pubblica e Zootecnia, Università degli Studi di Bari, Valenzano, BA, Italy; 2 Division of Parasitic Diseases and Malaria, Centers for Disease Control and Prevention, Atlanta, Georgia 30341-3724, USA

## Abstract

Nowaday, zoonoses are an important cause of human parasitic diseases worldwide and a major threat to the socio-economic development, mainly in developing countries. Importantly, zoonotic helminths that affect human eyes (HIE) may cause blindness with severe socio-economic consequences to human communities. These infections include nematodes, cestodes and trematodes, which may be transmitted by vectors (dirofilariasis, onchocerciasis, thelaziasis), food consumption (sparganosis, trichinellosis) and those acquired indirectly from the environment (ascariasis, echinococcosis, fascioliasis). Adult and/or larval stages of HIE may localize into human ocular tissues externally (i.e., lachrymal glands, eyelids, conjunctival sacs) or into the ocular globe (i.e., intravitreous retina, anterior and or posterior chamber) causing symptoms due to the parasitic localization in the eyes or to the immune reaction they elicit in the host. Unfortunately, data on HIE are scant and mostly limited to case reports from different countries. The biology and epidemiology of the most frequently reported HIE are discussed as well as clinical description of the diseases, diagnostic considerations and video clips on their presentation and surgical treatment.

*Homines amplius oculis, quam auribus credunt*

Seneca Ep 6,5

Men believe their eyes more than their ears

## Background

Blindness and ocular diseases represent one of the most traumatic events for human patients as they have the potential to severely impair both their quality of life and their psychological equilibrium. Although it is highly unusual, blindness has always been of great interest in human medicine. For example, the evaluation of the emotional and quality of life impacts in patients with some diseases causing blindness (e.g., macular degeneration) gave results similar to those found in diseases such as AIDS, chronic obstructive pulmonary disease, cardiac disorders and leukemia [1]. In addition, blindness has profound human and socio-economic consequences with high costs for the individual, and society, linked to lost productivity and rehabilitation estimated at $42 USD billion per year in 2000, and predicted to reach as high as $110 USD billion per year in 2020 [2].

There are many causes of blindness and those induced by parasitic agents (i.e., Protozoa, Helminths and Diptera) are of major public health concern in developed and developing countries. For example, eye disease caused by river blindness (*Onchocerca volvulus*), affects more than 17.7 million people inducing visual impairment and blindness elicited by microfilariae that migrate to the eyes after being released by female adult worms in the subcutaneous tissues [3]. Several parasites localize in human eyes as an effect of a specific neurotropism (e.g., *Toxoplasma gondii *in the foetuses), larval migration (e.g., ascarids, *Dirofilaria *spp., *Trichinella *spp.) and, in a few cases, as a primary localization being released directly into the eyes (e.g., *Thelazia callipaeda *eyeworm and some oestrid fly larvae causing myiasis) [4].

The present article focuses on those zoonotic helminths transmitted from animals to humans that affect the human eye. Undoubtedly, the parasitic zoonotic diseases and their epidemiology have been changing as a result of complex factors including abiotic (e.g., increasing temperatures) and biotic (e.g., demographical changes, political upheaval and land- use practices) that render this topic of great interest for the scientific community [5]. In addition, the impact of zoonotic diseases may vary in relationship to the socio-economic context and to the public health systems in different geographical areas [6], and for some infections, a greater threat exists for populations in developing countries [7]. Zoonotic helminths infecting eyes (HIE) include those transmitted by vectors (i.e., vector borne zoonosis, VbZ), by food consumption (i.e., food-borne zoonosis, FbZ) and those at direct transmission from the environment (i.e., water, soil, etc.) also known as environmentally-borne zoonosis (EbZ). A list of those helminths along with their route of transmission, geographical distribution, localization in the eye and definitive host species is provided in Table 1. Unfortunately, data on HIE are scant and mostly limited to case reports from different countries. Therefore a broad view of these infections on public health is lacking and ophthalmologists have difficulties managing HIE caused diseases and providing a clear diagnosis and therapeutic option for them. The present article focuses on those zoonotic helminths naturally infecting animals but which, occasionally, are transmitted to humans and affect the eye. Recent advances in the diagnosis and control of these parasitic infestations are also discussed on the basis of their distribution in different geographical areas and of interest in travel medicine.

**Table 1:** Classification (Order, Family and Species) of zoonotic helminths causing human blindness divided according their route of transmission (Vector borne zoonosis, VbZ, food consumption, FbZ, and those at direct transmission from the environment, EbZ), geographical distribution, localization in the eyes and zoonotic relevance.

OrderOrder (Family)	Species (common name)	Route of transmission	Geographical distribution	Localization	Definitive host	REF
**HELMINTHS**						

**Strongylida**						

	*Angiostrongylus cantonensis *(rat lungworm)	Ingestion of snails, slugs, shellfish and crustacean (FbZ)	Asia, Australia, Africa, USA, Pacific Islands, Caribbean Islands, and South America	Anterior chamber, vitreous	Rat	131,137

**Ascaridida (Ascarididae)**						

	*Toxocara canis *(dog roundworm)	Dog faeces (EbZ)	Worldwide	Eyebrows and eyelds, aqueous humor and vitreous	Dog	11

	*Toxocara cati *(feline roundworm)	Cat faeces (EbZ)	Worldwide	Aqueous humor and vitreous	Cat	11

	*Baylisascaris procyonis *(raccoon roundworm)	Raccoon faeces (EbZ)	North America, Europe, Japan	Vitreous	Raccoon	46

**Spirurida Suborder Spirurina**						

	*Gnathostoma spinigerum*, *G. hispidum*,	Ingestion of crustacean (cyclops),	Worldwide	Anterior chamber, eye lid	Dog, cat, wild carnivores,	49

	*G. doloresi *and *G. nipponicum*	infected fish, frogs (FbZ)			raccoon, opossum	

**(Onchocercidae)**						

	*Dirofilaria repens*	*Aedes*, *Anopheles*, *Culex *(VbZ)	Europe, Asia, Africa	Subconjunctival	Dog	8, 197

	*D. immitis*	*Aedes*, *Anopheles*, *Culex *(VbZ)	Worldwide	Anterior chamber	Dog, cat	8,76

	*D. tenuis*	*Aedes*, *Anopheles*, (VbZ)	Canada, USA	Subconjunctival	Raccon	8

	*Acanthocheilonema arbuta*	*Aedes*, *Taeniorhynchus *(VbZ)	Canada, USA	Anterior chamber	Porcupine	198

	*A. sprenti*	Mosquitoes (VbZ)	Canada, Oregon, USA	Anterior chamber	Beaver	8

	*A. reconditum*	Fleas, louses (VbZ)	Worldwide	Subconjunctival	Dog	79

	*Onchocerca gutturosa*	*Simulium *spp. (VbZ)	Worldwide	Subconjunctival, cornea	Cattle	8

	*O. cervicalis*	*Culicoides *spp. (VbZ)	Worldwide	Subconjunctival, cornea	Horse	169

	*Onchocerca jakutensis*	*Culicoides *spp. (VbZ)	Europe, Austria	Subconjunctival, cornea	Red deer	199

	*O. reticulata*	*Culicoides *spp. (VbZ)	Europe, Asia, Africa	Subconjunctival, cornea	Horse	170

	*O. dewittei japonica*	*Simulium *spp. (VbZ)	Japan	Subconjunctival, cornea	Bear	167

	*O. lupi*	*Simulium *spp. (VbZ)	Europe	Subconjunctival	Dog	172

	*Loaina uniformis*	*Aedes*, *Anopheles*, *Culex*	North America	Subconjunctival	Lagomorphs	8,62,64,83

	*P*. (*Loaina*) *scapiceps*	*Aedes*, *Mansonia*	North America	Subconjunctival	Lagomorphs	8,62,83

**(Thelaziidae)**						

	*Thelazia callipaeda T. californiensis*	*Phortica *spp. (VbZ)	China, Southeastern Asia, Europe, USA	Subconjunctival, intraocular	Dogs, cats, rabbits and wild carnivores	160

**Enoplida**						

(Trichuridae)	*Trichinella *spp.	Ingestion of raw meat (FbZ)	Worldwide	Orbit, Ocular muscles	Numerous, domestic and wild animals	15

**CESTODA**						

**Pseudophyllidea (Diphyllobothriidae)**						

	*Spirometra erinaceieuropaei *(Sparganosis)	Ingestion of crustacean, frogs, birds, snakes (FbZ)	Middle East, Australia	Subconjunctival	Carnivores	102,109

	Spargana (other species)	Ingestion of crustacean, frogs, birds, snakes (FbZ)	South America, Asia	Subconjunctival	Carnivores	102,109

Cyclophyllidea (Teniidae)						

	*Taenia crassiceps*	Food contaminated by dog faeces (EbZ)	USA and Europe	Anterior chamber	Carnivores	89

	*Echinococcus granulosus*	Food contaminated by dog faeces (EbZ)	Worldwide	Intraocular	Dog	112

	*E. multilocularis*	Food contaminated by wild carnivores dog faeces (EbZ)	Worldwide	Intraocular	Wolf, jackal, coyote	115

	*E. oligharthrus*	Food contaminated by wild carnivores dog faeces (EbZ)	South and Central America	Orbit	Wild felids	Wild felids

	*Coenurus cerebralis *(*Multiceps multiceps*)	dog faeces (EbZ)	Worldwide	Intraocular	Dog	95

**TREMATODA**						

**(Fluke)**						

	*Fasciola hepatica*	water plants (FbZ)	Worldwide	Anterior chamber	Domestic and wild ruminants, horse	120

	*Alaria *mesocercaria, *A. americana*	Frogs (FbZ)	Asia, USA, Canada	Intraocular	Canids	122

	*Philophthalmus lacrimosus*	Contaminated food or direct contact with the eye mucosa (EbZ)	Europe, Asia, and America	Conjunctival	Birds	124

## Biology and pathogenic effects

Helminths at the adult and/or larval stages may infect human ocular tissues externally (i.e., eyelids, conjunctiva sacs, subconjunctiva, and lachrymal glands) or the ocular globe (i.e., optical nerve, intravitreous retina, anterior and posterior chamber). Several parasitic helminths adapted a tropism for animal eyes and related tissues when migrating throughout the host body mainly during their immature stages. This is the case of ascarids and strongylids, causing ocular larva migrans, filarioid species, and larvae of *Trichinella*, as well of trematode and cestode parasites. Nonetheless, human ocular infestations by zoonotic helminths may also be caused by the parasitic adult stages as in the case of thelaziids (eye worm infestation) and filarioid species including those belonging to the genera *Dirofilaria *and *Onchocerca *[8-10]. Thus, ocular localization of helminths is mainly caused by aberrant migration in host tissues and, only in one case (i.e., *T. callipaeda*), by direct inoculation into the eyes. What is equally unclear is the route that most follow to gain entry into the eye. It is supposed that some migrate along and follow the optic nerve but others may enter the bloodstream and be carried to the eye in that manner; however, it is not known if these are the preferred or aberrant routes, or even which is the most common route that helminths follow to reach the eye. Once in the eye, larvae likely find it to be a more protected site from host immune responses, but it is not clear that a directed migration into the eye had occurred.

Consequently, ocular alterations caused by zoonotic helminths vary considerably causing mild to severe clinical signs, including lacrimation, epiphora, conjunctivitis, keratitis, corneal ulcers, or retinal lesions, resulting in vision loss (Table 2). For example, in ascarid infections, visual impairment or blindness results from larval migration, with destruction of the visual cortex. In addition, larvae might develop inside the patient's eye (e.g., in baylisascariasis) progressively impairing the vision. However, blindness might be also an effect of the immune reaction the parasites elicit in the host body, or of a combined effect of both presence of the parasite and antibody-mediated reaction. This is the case of ascarids in which ocular signs are related to inflammation because of the presence of larvae and a local immune reaction to them in the retina [11,12].

**Table 2:** Ocular tissue affected and symptoms caused by zoonotic helminths (Genus and/or Species) at different stage [41,63].

Ocular tissue affected	Signs	Helminths involved
Eyebrows and eyelids	Eye lid edema	*Taenia solium *(cysticercus), *Spirometra*, *Ancyclostoma*, *A. Americana Gnathostoma*, *Toxocara*, *Trichinella*, *Dirofilaria*

Lacrimal duts and glands	Lacrymation	*Mammomonogamus*, *Thelazia*

Orbit	Exophthalmos	*Echinococcus*, coenurus, *Taenia solium *(cysticercus), spargana, *Trichinella*, *Dirofilaria*, *Gnathostoma*

Ocular muscles	Diplopia	*Trichinella*, *Angiostrongylus*, *Ancyclostoma*, spargana, *Taenia*

**Conjunctiva**	Subconjunctival cysts	*Taenia, Dirofilaria, Acanthocheilonema, Habronema, Mansonella*, spargana, *Philophthalmus*
	Chemosis and conjunctivitis	*Thelazia, Trichinella, Onchocerca*
	Hemorrhages	*Trichinella*

**Cornea**	Keratitis, scleritis	*Onchocerca, Toxocara, Ancylostoma*

Anterior chamber	Parasites in the anterior Chamber	*Onchocerca, Schistosoma, Taenia, spargana, Angiostrongylus, Gnathostoma, Toxocara, Dirofilaria, Thelazia, Acanthocheilonema*
	Cysts in the anterior chamber	*Taenia*
	Hypopyon	*Taenia, Gnathostoma, Toxocara*
	Secondary glaucoma	*Taenia, Echinococcus, Angiostrongylus, Dirofilaria, Onchocerca *spp., *Gnathostoma, Toxocara*

Iris	Mydriasis	*Trichinella*
	Miosis	
	Distortion of the pupil	*Onchocerca*
	Iritis and iridocyclitis	*Taenia, Angiostrongylus, Ancyclostoma, Trichinella Toxocara, Onchocerca, Pelecitus*

Vitreous body	Hemorrhages	*Trichinella, cysticercus, Gnathostoma*
	Cysts	*Cysticercus, Echinococcus, coenurus*
	Parasites in the vitreous Cyclitis	spargana, Acanthocheilonema, Dirofilaria, Onchocerca Gnathostoma, Onchocerca, Toxocara, Trichinella

Optic nerve	Papilledema, papillitis, and optic atrophy	*Taenia, Ancylostyoma, Toxocyara, Trichinella, Onchocerca*

Retina and chorioidea	Hemorrhages	*Ancylostoma, Gnathostoma, Toxocara, Trichinella*
	Retinal detachment	*Taenia*
	Cysts	*Echinococcus*
	Retinitis and choroiditis	*Baylisascaris, Taenia, Toxocara, Trichinella, Onchocerca*

## The most commonly reported HIE

### Nematodes

There are many nematode parasites that can be found in the orbit or within the eye proper (Table 1). Although most nematode infections of the eye are rare, some are more frequently reported than others. In this section, we will discuss those zoonotic nematodes that are most likely to be encountered and reported, by examining their aetiology, case reports and epidemiology.

#### Trichuroids

Trichinellosis (Trichuroidea, Trichinellidae) has a cosmopolitan distribution, but is generally less important as an infection of humans in the tropics than in more temperate regions of the world. Once thought to be a single species (*Trichinella spiralis*), there are now at least eight distinct species recognized. Each of these species has a slightly different geographical distribution and host range, and only *Trichinella zimbabwensis *of crocodiles in Tanzania, has not been reported from humans to date. The most striking feature of this group of parasites is their obligatory transmission by ingestion of infected meat containing larvae, either in typical cysts or unencapsulated in the case of several species [13]. The clinical course is characterized by two phases, the enteric/enteral phase, when adult worms are present in the intestinal mucosa, and the parenteral phase, when the released larvae invade the host muscles [14]. During the parenteral phase, which follows the enteral, a typical syndrome of fever, myalgia, periorbital edema and eosinophilia occur. In addition to periorbital and facial edema, conjunctivitis is also frequent. The cause of the orbital and facial edema is not well known. but probably includes some component of an allergic response. Periorbital edema often appears early in the parenteral phase, and typically begins to wane after several weeks. Larvae also affect the macula and retina, causing hemorrhage and other damage as they migrate through and into these ocular tissues [15]. The diagnosis can be suggested from clinical history of ingesting raw or inadequately cooked meat, and the ophthalmologist is often the first contact because of the swollen eyelids and conjunctivitis. Demonstration of larvae in muscle biopsies of patients or in frozen sample of the ingested meat, if available, is still standard procedure to confirm infection. Good serological tests exist and are also very useful in confirming infection, especially in cases with low-level infection where symptoms may be minimal and the number of larvae in muscle may be low. Some areas of the world, such as the United States and Europe, have effectively controlled the infection in humans by removing the parasite from the domestic pig cycle through heightened food safety regulations regarding inspection and feeding practices. In other areas of the world, the domestic pig cycle continues to be responsible for human infection, and in all areas, human infection continues to occur when infected wild game meat is ingested without proper cooking. Because of the wide range of animals that can harbour infection with *Trichinella *larvae, proper handling and cooking of all meats is recommended.

#### Ascarids

Ascarids (Ascaridida, Ascaridiidae) occur worldwide infecting various mammals, including humans [16,17]. Many of these nematodes are causative agents of zoonoses transmitted to humans via contaminated soil. Within the ascarids, *Toxocara canis, Toxocara cati *and *Baylisascaris procyonis *are zoonotic parasites of dogs, cats and raccoons, respectively, and they are among the most widespread causes of neural and ocular larva migrans. Indeed, larvae of *T. canis *are probably the most common nematode infection of the human eye, also known as ocular larva migrans (OLM), and infection in humans occurs worldwide [11]. Infection occurs through the ingestion of infective eggs, most often from soil or other environmental surfaces that have been contaminated with faeces from infected animals. Examination of soil or sand from parks and playgrounds often demonstrates infective *Toxocara *eggs, which might remain infectious for long periods of time (even years) in the environment [18]. When ingested, the eggs hatch and larvae migrate in the tissues, most often to the liver, but on occasion to other sites such as the eye and central nervous system (CNS). The wandering larvae cause a syndrome, called visceral larva migrans (VLM), of marked eosinophilia, hepatomegaly, fever, cough, and pulmonary infiltrates. The severity of symptoms is often related to the number of larvae acquired, and can range from asymptomatic to acute, with a fatal outcome. The ability of *Toxocara *larvae to cause OLM was recognized about 60 years ago [19,20]. OLM occurs most typically in older children (mean 8 yr versus 2 yr for VLM), generally have no other evidence of organ involvement, and hypereosinophilia, hepatomegaly, and pulmonary symptoms are absent, there is no history of pica, and evidence suggests that OLM is caused by a single larva entering the eye. Antibodies to *Toxocara *tend to be lower in cases of OLM, possibly as a result of fewer infective larvae, and there is experimental evidence that somewhat different immune responses occur between OLM and VLM [21]. Hundreds of cases have been reported and described and untold thousands of cases have probably occurred, even in developed countries, as evidenced by seropositivity in population-based surveys [22,23]. Worldwide, cases continue to be reported in the literature, including descriptions of lesions, effective treatments, and new/modified methods to observe the infection in the eye [24-37]. Visual observation of motile larvae in the eye is possible, although accurate diagnosis is difficult; serodiagnosis continues to be very useful in detecting and confirming cases [38,39]. *Toxocara *larvae are approximately 400 by 20 μm and a larva of this size in the eye is highly suggestive. In this presentation, destruction of the larva by photocoagulation is recommended, and prognosis is favourable when recognized early and prompt treatment is provided [40]. After an undefined period of wandering in the tissues, but probably for several weeks or longer, larvae become encapsulated, including those in the eye. These cases, typically present with unilateral visual deficits, with or without ocular pain, and a raised white retinal mass that presents difficulty in distinguishing from retinoblastoma. Unfortunately, in these situations, loss of visual acuity, blindness, and even enucleation of the eye may result. *Toxocara *larva seen in biopsy specimens or surgically resected tissues are rather easily identified based on size and morphological features. Generally the larva will be enclosed in a granuloma, coiled, and one or more sections of the larva evident. In tissue sections, larvae measure 15-21 μm in diameter and are characterized by a single prominent lateral ala, non- patent gut, and large excretory columns [41]. The prevention of toxocariasis, including OLM, is based on good personal hygiene, including washing hands, and the proper disposal of pet waste, including and specifically not letting pets and stray animals defecate in public places where children and others play and could come in contact with infective eggs. Rubinsky-Elefant and colleagues [11] recently reviewed the subject.

**Figure 1:**
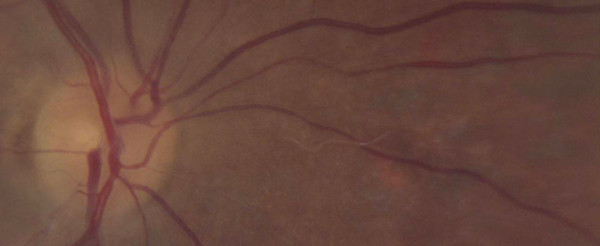
**Nematode larva in the retinal fundus**. Larva, presumably of *Baylisascaris procyonis*, in the retinal fundus of a patient. The infection was presumed to have been acquired in Connecticut, USA. Larva measures approximately 1.4 mm and was treated with retinal lasar. (Original; courtesy of Drs. Caplivski, Bhatnagar, and Goldberg, Mount Sinai School of Medicine).

Ocular disease in baylisascariasis occurs in association with severe neural and VLM and, only rarely, alone. Among the ascarids, *Baylisascaris *species are most often implicated in serious cases of neural and OLM [42-45]. *Baylisascaris procyonis*, a common ascarid of raccoons in many parts of the United States, Europe, and Japan, has demonstrated potential for extensive larval migration in rodents and birds or other accidental hosts where it can produce a fatal eosinophilic meningoencephalitis. This nematode, different from other causes of larva migrans, has an aggressive somatic migration with larval invasion of the central nervous system and capability for continued larval growth within intermediate hosts [46]. Recent serological studies have indicated that significantly more exposure to this parasite is occurring than previously thought, and consequently, low level infections may also be more common than believed [46]. As we recognize increased exposure, the potential exists for more cases of serious visceral and CNS disease, including migration into the eye (Figure 1). Baylisascariasis is acquired in the same manner as other ascarids, through the ingestion of infective eggs from the soil or other environmental sources that have been contaminated by raccoon faeces. Similar to other visceral or OLM, cases of baylisascariasis undoubtedly span the spectra of asymptomatic to serious, often fatal infections. *Baylisascaris *spp. differ from *Toxocara *spp. in that the larvae continue to grow, often reaching 1-2 mm in length (by 50 - 60 μm in diameter) in the tissues, including the eye. The migration of such relatively large larvae can result in significantly more pathology than that of *Toxocara*. The main symptoms are represented by chorioretinitis, optic neuritis, or optic nerve atrophy (Table 2) and examination occasionally may reveal motile larvae migrating within the retina [11,47] and vitreous humour [48]. Ocular signs are related to the inflammation and local immune reaction of the retina, retinal vasculature, and optic nerve to the larvae. Prevention of baylisascariasis is directed at avoiding ingestion of infective eggs. This is best accomplished by reducing the environmental contamination with raccoon faeces through not feeding wild animals and not encouraging them to live in close proximity to humans, prompt and safe clean up of raccoon faeces/latrines, and not letting children play in areas that have been contaminated with raccoon faeces [46].

#### Spirurids

##### *Gnathostoma *spp.

**Figure 2:**
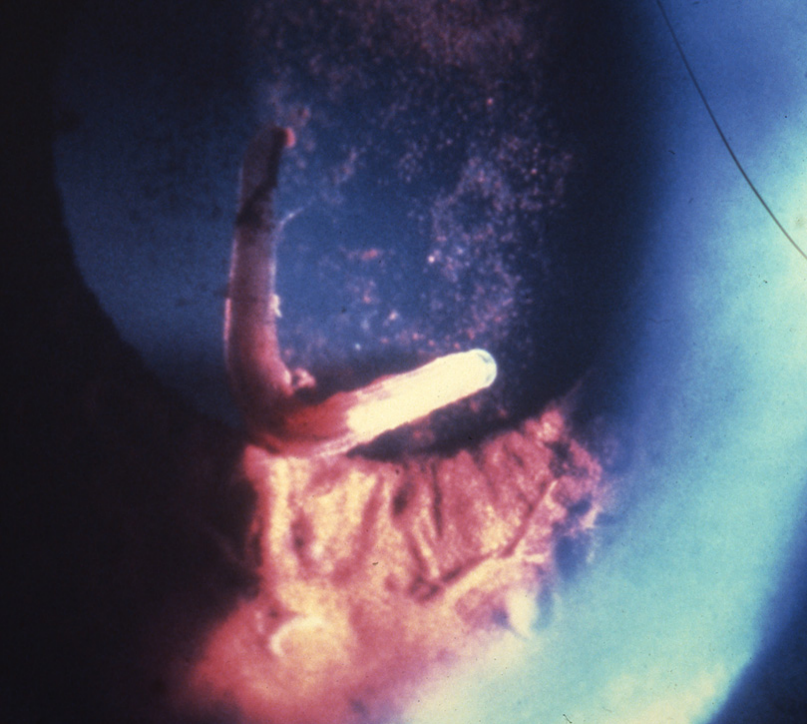
***Gnathostoma spinigerum *larva in the anterior chamber of the eye**. *Gnathostoma spinigerum *larva in the anterior chamber of the eye of patient in Thailand. (From Teekhasaenee C, Richt R, Kanchanaranya. **Ocular parasitic infection in Thailand**. *Rev Inf Dis*. 1986, 8:350-356).

Another form of larva migrans is caused by spirurid worms in the genus *Gnathostoma *(Spirurida, Gnathostomatidae) [49]. The definitive hosts include dogs, cats, wild carnivores, raccoons and otters. Due to the large range of intermediate (e.g., fish, frogs, chickens and other birds, snakes, pigs, lizards, some crabs, monkeys, hamsters, rats, mice, squirrels, and guinea pigs) and paratenic (birds, snakes, and frogs) hosts, the control of this parasitic infection is particularly difficult. The zoonotic infections occur much more commonly as cutaneous larva migrans (CLM) or VLM, but on rare occasions can invade the eye [50-59]. Ocular gnathostomiasis can involve both invasion of surrounding tissues or the eye itself by a wandering larva. In the former, edema and hemorrhage of the eyelid or inflammation of the orbit may occur. When a larva enters the tissue of the eye, corneal ulceration, iris perforation or retinal artery occlusion may occur, with pronounced uveitis, vitritis, vitreal hemorrhage, or secondary glaucoma. Infections are acquired through accidental ingestion of infected copepods that harbour 2^nd ^stage larvae, but more often through the ingestion of poorly-cooked infected fish or other paratenic hosts such as frogs, snakes, birds or even other mammals. Migration of larvae directly from infected animal tissues used as poultices may also occur and place the larvae in the immediate vicinity of the eye. Four species of *Gnathostoma *have been reported from humans, including * Gnathostoma spinigerum*, *Gnathostoma hispidum*, *Gnathostoma doloresi*, and *Gnathostoma nipponicum*. However, many other species occur in nature and may pose a threat of zoonotic infection to man. These are relatively stout larvae, and the 3^rd ^stage larva in human tissue can measure between 1 and 5 mm or more in length by 200-600 μm in diameter. The larval stages have many morphological features in common with adult worms, including the prominent head bulb and cuticular spines. In sectioned tissue, the size of the larva is again distinguishing, and occasionally both the head bulb and cuticular spines may be seen, but are absent in many sections. The cuticle can vary between thin and thick, and the muscle cells are numerous and well defined; the lateral chords tend to be large and very prominent, and the gut is distinctive in nature, being composed of many cuboidal cells, each with multiple nuclei, and a luminal brush border microvilli [41]. The intestine and its lumen can be round or variously shaped but is generally a prominent structure noted inside the larva. Serology has proven useful in diagnosing cases [49,60,61]. Because of the size of the larva, and their ability to migrate readily in the tissue, its location within the eye is generally of concern, although there are reports of successful recovery of larva from the eye with restoration of visual acuity (Figure 2) [50]. Prevention is primarily through the avoidance of eating poorly-cooked foods or use of raw flesh as poultice.

#### Filarioids

There are a number of different filarioids that have been reported infecting the eye or the conjunctiva, and those reports date back several hundred years, making them one of the oldest groups of parasites known to occur in or on the eye. Indeed, besides the well-known (but not zoonotic) *Wuchereria bancrofti*, *Brugia malayi *and *Loa loa*, some filarioids from domestic and wild mammals (e.g., *Dirofilaria *spp., *Onchocerca *spp., *Acanthocheilonema *(*Dipetalonema*) spp., *Brugia *spp., and *Loaina *spp.) have a zoonotic origin and may infect human eyes [8,62,63]. In addition, a number of yet incompletely identified filarioids have been described in human eyes in the Amazon forest regions [64,65]. For many of them, the life cycle and animal reservoir hosts are poorly known. Animal filarioids occur globally, in many different forms, and all filarioid infections are transmitted by various bloodsucking arthropods; the majority of them, including zoonotic infections, by mosquitoes, although blackflies, culicoids, and others may be involved. Most persons worldwide are at some risk, and those who are more likely to be exposed to the vectors may be at increased risk, but given the worldwide occurrence of animal filaria, there are probably other undefined risk factors.

The largest number of filarioid eye infections is caused by species of *Dirofilaria *and these result in a worm that migrates across the conjunctiva or is encapsulated in a nodule on the conjunctiva or eyelid. These cases were referred to as *Filaria conjunctivae *[66], for decades until they were properly aligned with known animal species [67]. Hundreds of these cases have been reported and new cases continue to be reported from wide geographic areas, including areas that have not previously reported such cases [68-76]. Undoubtedly, many more cases occur and are either not recognized or reported. In the United States, these are most often caused by *Dirofilaria tenuis*, a common parasite of raccoons, and in Europe and other parts of the world, by *D. repens*, a common parasite of dogs and other canids (See additional file 1: Movie1 Surgical removal of *Dirofilaria repens *from patient's conjunctiva) [77]. Other *Dirofilaria *species, such as *Dirofilaria ursi *of bears, *Dirofilaria subdermata *of porcupines, *Dirofilaria striata *of wild cats, and others have been isolated from humans on occasion [8]. These species have not been reported to involve the eye, but they certainly could in the future. In worms removed intact or broken from the conjunctiva or seen in histological sections of nodules, the morphologic features of most *Dirofilaria *make them relatively easy to identify to genus level [8]. They tend to be large, robust worms, and they have distinctive longitudinal and circular cuticular ridging that gives the external cuticle a beaded or corn-row appearance. This can be seen easily in gross specimens that have been removed intact, and is one of the more prominent features noted in histological section as well. Additionally, in sections, the worms have numerous strong muscle cells (polymyarian and coelomyarian) giving a strong body wall. Determining the species is more difficult, especially if a male worm is not present, and final diagnosis is often based on the presumed location of acquisition (i.e., *D. tenuis *if in the United States, *D. repens *elsewhere). Once removed, clinical signs quickly resolve and there are no residual sequelae.

Worms identified as *Dirofilaria *have also been reported from within the eye, either the anterior chamber or vitreous. Some of these cases have been attributed to *D. immitis*, the dog heartworm, *D. repens*, or *Dirofilaria roemeri *of kangaroos [8]. A small number of them were successfully removed and identified based on morphology. A case of intravitreal dirofilariasis was recently reported from Turkey [78] and a case of human intraocular dirofilariasis has been reported from northern Brazil (See additional file 2: Movie2 Surgical removal of *Dirofilaria immitis *like nematode) [65]. The nematode from Brazil was morphologically and phylogenetically close to *D. immitis *but genetically distinct from reference sequences, including those of *D. immitis *collected from infected dogs in the same area. The possible existence of a closely related zoonotic *Dirofilaria *species in Brazil and its implications have been discussed and serve to highlight the high number of yet unknown species infecting wild mammals that have potential to cause zoonotic infections. Different from the above reported case, worms were generally of modest size, making removal rather than photocoagulation the preferred method of treatment. *Dirofilaria *spp. worms are often motile, and noticed by the patient because of interference with vision. Removal is curative and full visual acuity is generally restored with no long term sequelae.

**Figure 3:**
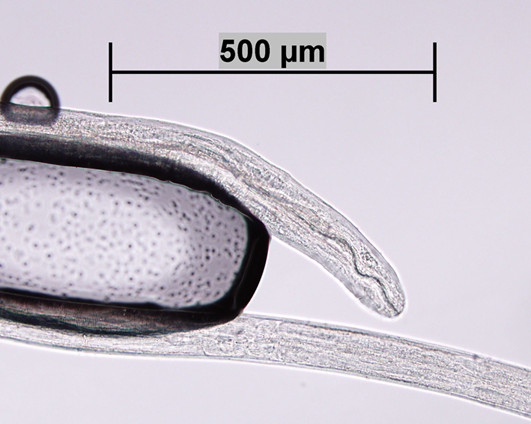
***Acanthocheilonema *- *Mansonella *-like worm**. Anterior end of an *Acanthocheilonema - Mansonella *-like worm removed from the posterior chamber of the vitreous of a patient in Kansas, USA. Scale bar = 500 μm. Original; courtesy of DPDx, CDC.

In addition to *Dirofilaria*, several cases of small *Acanthocheilonema *-like worms within the eye have also been reported (Figure 3) [63]. Although removed and examined, it was not possible to positively identify these worms to species. In a single case report, *Acanthocheilonema *(*Dipetalonema*) *reconditum*, a subcutaneous filarial infection in dogs worldwide, was removed from the subconjunctiva in a patient from Australia [79]. Two other unusual infections caused by *Macacanema*, a parasite of monkeys, have been reported where worms were removed from the conjunctiva of humans [80,81].

The zoonotic role of filarial infection in humans in some regions is far from understood. Recently, a mature male filaria extracted from the iris fibers of a man from the Amazon region of Brazil was identified as belonging to the genus *Loaina *or *Pelecitus *(See additional file 3: Movie 3 Surgical removal of *Pelecitus *sp. from the iris fibers of a patient) [64]. This human case and a previous one from Colombia [62] were of unknown origin and both occurred in the tropical Amazon region but little is known about the source of the infection. Vectors of species of *Loaina *and *Pelecitus *are mosquitoes, mallophagans or tabanids, as shown with the three cycles elucidated [82-84]. Unfortunately, despite some studies on these genera [85,86], information on these taxa is scant. In the same manner, worms removed from the eye and identified as *Brugia *are often hard to identify as *B. malayi *of human origin or *Brugia *spp. of animal origin [87].

### Cestodes

#### *Taenia crassiceps*

**Figure 4:**
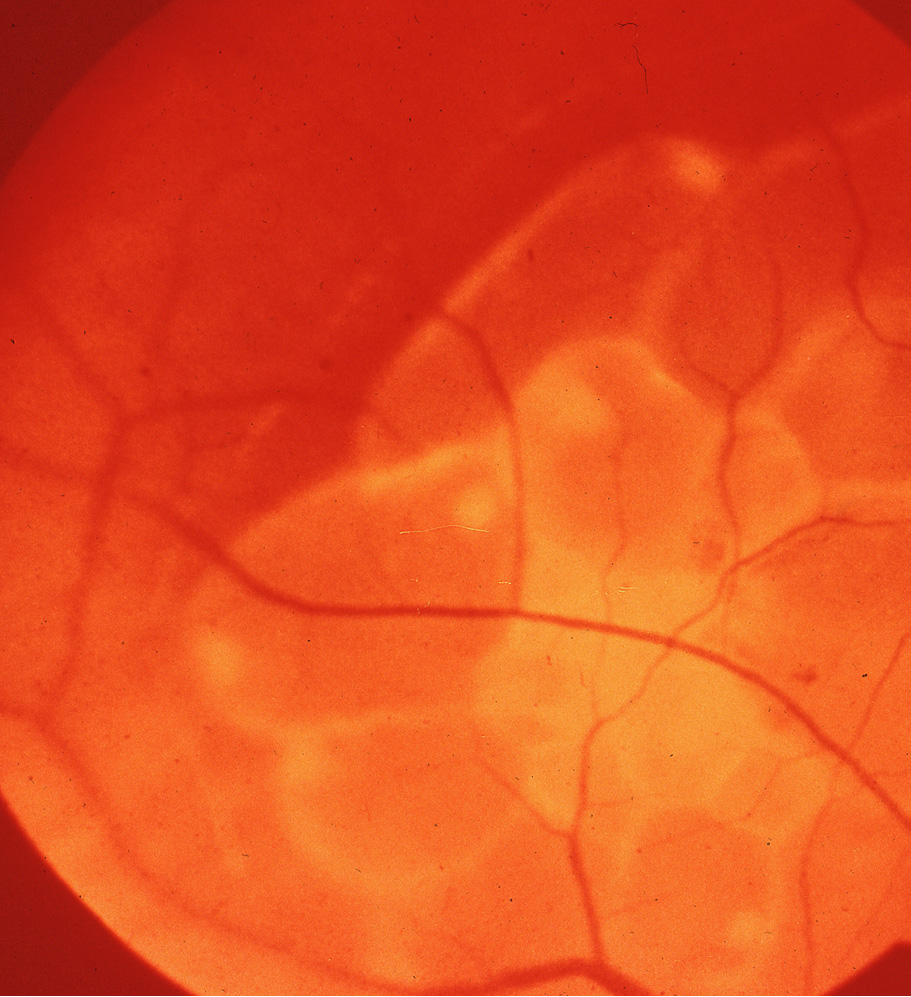
**Cysticercus cyst of *Taenia crassiceps *in situ**. Retinal photograph showing budding cysticercus of *Taenia crassiceps* in situ. (From the collection of Herman Zaiman, "A Presentation of Pictorial Parasites").

Human ocular infection by *Taenia crassiceps *occurs when individuals accidentally ingest eggs in contaminated food or water. Indeed, *T. crassiceps *is a tapeworm closely related to *Taenia saginata *and *Taenia solium *and adult stages live in the intestine of carnivores and pass eggs with the faeces. In the intermediate hosts, primarily rodents, the immature cestodes (i.e., cysticerci) develop in the peritoneal cavity [88]. In humans, larvae invade the bloodstream and reach subcutaneous and muscular tissues of immune- compromised individuals [88,89]. Interestingly, larval *T. crassiceps *in the anterior eye chamber or subretinally (Figure 4) have been reported in immunocompetent humans, in the United States [90,91] and Europe [92]. The infection by cysticerci of *T. crassiceps *may be asymptomatic or cause iridocyclitis and/or retinitis [88,92]. Surgical intervention on the anterior chamber or the subretinal space is successful in curing the infection [88,92]. Although not a zoonosis, cysticercosis caused by *Taenia solium*, has been reported from the human eye [93].

#### *Coenurus cerebralis*

**Figure 5:**
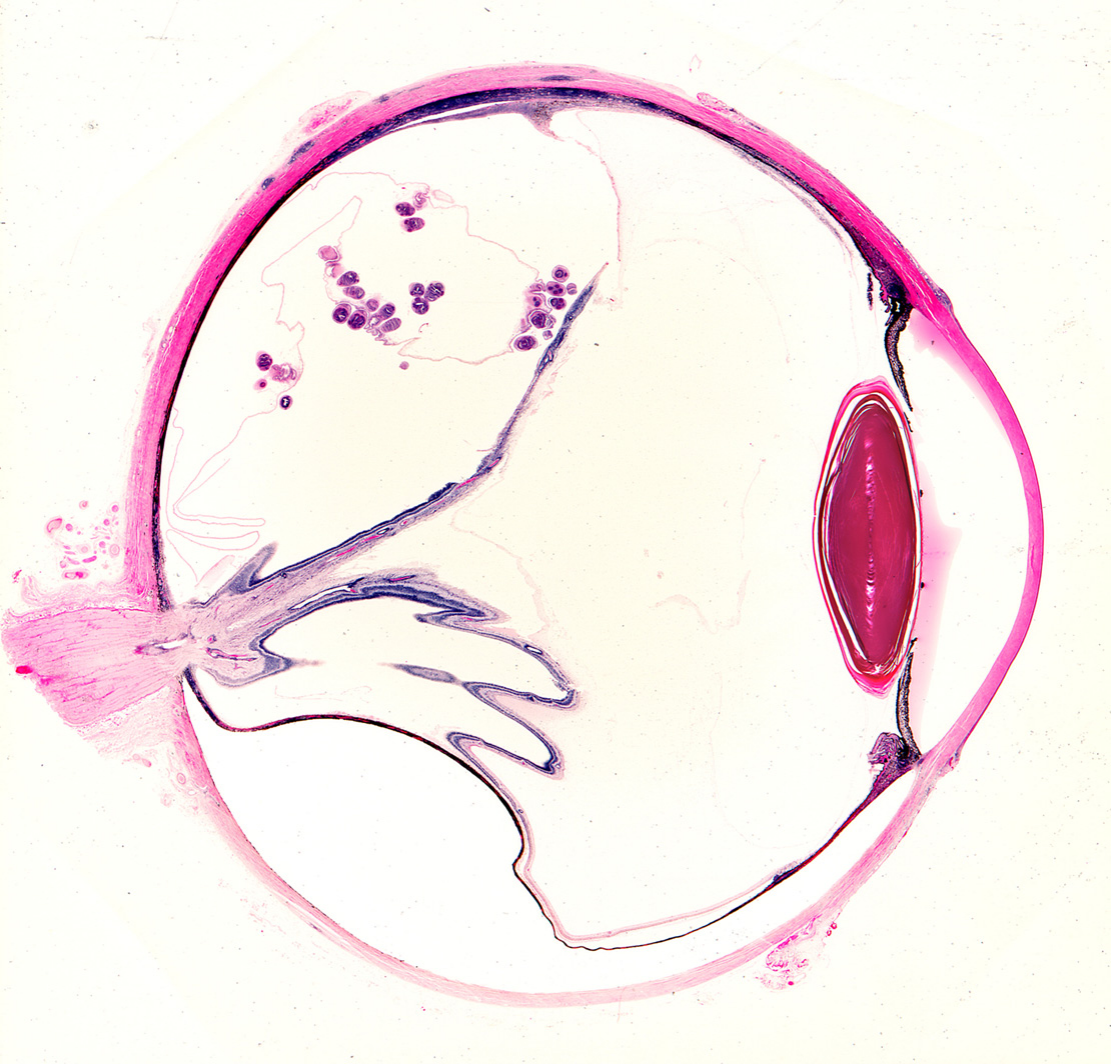
**Coenurus cyst behind displaced retina**. Sagittal section of eye from Ghanaian showing coenurus cyst with multiple protoscoleces lying behind displaced retina. (From Parasites In Human Tissues, Orihel and Ash, ASCP Press, 1995).

**Figure 6:**
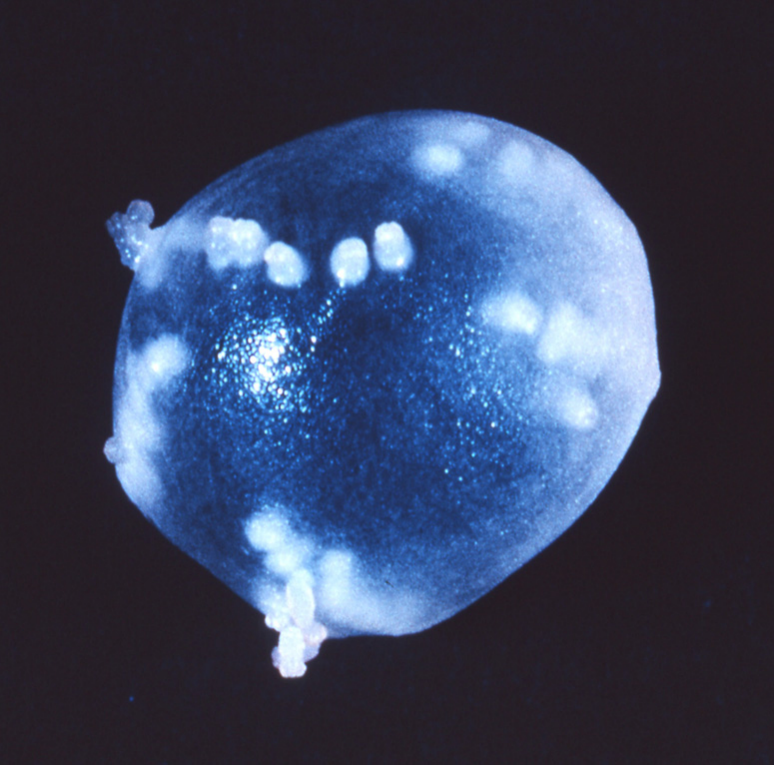
**Coenurus cyst after surgical removing from the eye**. Intact coenurus cyst removed from subconjunctival tissue an Ugandan child showing multiple protoscoleces. (Original by Paul Beaver).

*Coenurus cerebralis *is the larval stage of the tapeworm *T. multiceps *(syn. *Multiceps multiceps*), which develops in the small intestine of dogs, foxes, and other canids (definitive hosts). After ingestion by the intermediate hosts, the oncospheres penetrate the intestinal mucosa, enter the bloodstream, and reach the brain where they develop into the infective cystic coenuri [94,95]. Coenurosis rarely occurs in humans through accidental ingestion of eggs, causing mainly cerebral lesions but also localizing in the eyes [92]. *C. cerebralis *ocular lesions cause severe anterior uveitis, retrolental or orbital cystic tumor-like masses, and subretinal lesions (Figure 5). Subconjunctival localization may also occur after accidental direct inoculation with infective eggs. The onset of inflammatory responses result in a red and painful eye, followed by development of glaucoma, retinal fibrosis, and ultimately blindness as the final result of the infection [96,97]. Surgical removal of accessible cysts (Figure 6). is the only choice to cure the infection [96,98].

#### *Spirometra *spp.

Analogously, adult *Spirometra *cestodes live in the small intestine of carnivores where they release eggs which reach the environment with the host faeces. Larvae of *Spirometra *spp. tapeworms infect domestic animals and humans. Humans are dead-end hosts given that they become infected mostly by drinking polluted water (via ingesting the immature procercoid), or eating inected intermediate hosts (i.e., frogs, birds, snakes, that, along with rats and mice are infected with the larval stages) and assuming the plerocercoid larvae. Once ingested, the larvae ("spargana") may invade muscles, subcutaneous tissue, urogenital and abdominal viscera, and, sometimes, the central nervous system and the eyes [99]. Human ocular sparganosis has been reported from South America [100], Central Europe [101] and Asia [102-105]. Spargana usually infect subconjunctival and conjunctival tissues causing symptoms varying from simple itching due to local granulomata to more serious signs represented by local pain, epiphora, chemosis, and ptosis [105,106]. Conjunctival infection may also be characterized by irritation, continued foreign body sensation, redness [104] and mimic signs and symptoms of orbital cellulitis, with exophthalmia and corneal ulcers. When the immature cestode invades the orbit it may cause acute anterior uveitis and iridocyclitis [101] and severe inflammation with blindness [107]. Unfortunately, surgery is the only effective treatment [104,105].

#### *Echinococcus granulosus*, *Echinococcus multilocularis* and *Echinococcus oligarthrus*

*Echinococcus granulosus*, *Echinococcus multilocularis *and *Echinococcus oligarthrus *are tapeworms that occur worldwide. Adult stages of *E. granulosus *and *E. multilocularis *infect mainly dogs or wild canids (e.g., wolves, jackals, coyotes and foxes) while *E. oligarthrus *adults infect wild felines [108-111]. Along with several other animal species, human may act as (accidental) intermediate hosts of these cestodes by ingesting food contaminated by their eggs [110,111]. When a human being inadvertently ingests eggs, the larvae hatch and disseminate via the bloodstream into different organs and viscera (mostly liver or lungs but also heart), where they produce a typical hydatid cyst (*E. granulosus*, *E. oligarthrus*) or many alveolar small cysts (*E. multilocularis*) causing a major zoonotic disease [108-111].

Although not very common, ocular infection by larval *Echinococcus *spp. may thus occur as a consequence of bloodborne dissemination of the oncospheres. Ocular localization by the larval form of *E. granulosus *accounts for 1 to 2% of all reports. Intra-orbital hydatid cysts by *E. granulosus* may cause severe exophthalmia [112] pain and blindness as the hydatids have the ability to fill the vitreous cavity [113] or severe inflammation of orbital structures and acute eyesight loss due to the rupture of intraorbital hydatids [114]. Ocular alveolar hydatidosis caused by *E. multilocularis *may occur after spreading of the larval cestodes to other sites. For instance a choroidal eye mass has been reported in a patient with history of visceral alveolar hydatid disease with cerebral metastasis [115]. Human infection by *E. oligarthrus *is very rare with only a few cases published in the international literature, two of which involve the eye [116]. Nonetheless, the ocular localization of *E. oligarthrus *has a relevant clinical impact since it causes the presence of a single orbital, retro-ocular cyst in the orbit [117] or the occurrence of a retroocular cystic tumor-like mass inducing exophthalmia, chemosis, palpebral ptosis, and blindness [108].

### Trematodes

Fascioliasis, also known as liver fluke, is caused by *Fasciola hepatica *and *Fasciola gigantica*, trematodes which localize in the biliary ducts of the definitive hosts, grass-grazing domestic and wild ruminants (i.e., cattle, sheep, goats, buffaloes) and also horses and rabbits. This parasite develops through various larval stages in water snails of the genus *Limnaea *which release cercariae that encyst as metacercariae on aquatic vegetation. Infection occurs when animals ingest freshwater plants or water containing encysted metacercariae [118]. Within the last decade, reports of human cases of fascioliasis have increased [119]. Although migrating immature *F. hepatica *flukes in humans have been mainly reported in blood vessels, lung, subcutaneous tissue, and ventricles of the brain [119], they have also been recovered from the anterior chamber of a patient in Iran [120].

**Figure 7:**
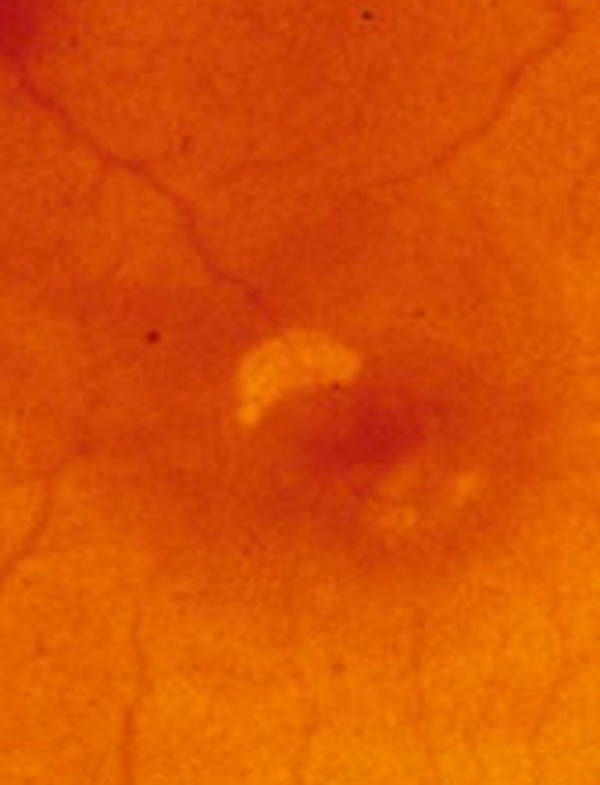
***Alaria *sp. in the eye**. Freely moving *Alaria *mesocercaria on the retina of the eye. The anterior sucker is evident on the left side of the organism. (From the collection of Herman Zaiman, "A Presentation of Pictorial Parasites").

*Alaria americana *(syn. *canis*) is a three-host trematode that lives as adults in the intestine of the dog definitive host. Eggs are passed in faeces and hatch in water, releasing miracidia which penetrate the helisomid snails (first intermediate host) and develop through the sporocyst stage into cercariae [121]. Cercariae released from snails actively penetrate the second intermediate host (tadpoles) becoming infective mesocercariae in about two weeks. In the tadpole or in the frogs (following the metamorphosis), mesocercariae accumulate and may be ingested by a number of paratenic hosts (e.g., other frogs, snakes) or directly by the definitive host. Cases of human intraocular infection with mesocercariae of *A. americana *and other *Alaria *mesocercariae have been recorded in patients who had ingested undercooked contaminated frogs legs [122]. Both patients presented with pigmentary tracks in the retina, areas of active or healed retinitis and signs of diffuse unilateral subacute neuroretinitis (Figure 7).

The trematode *Philophthalmus lacrimosus *(Philophthalmidae), as adults, parasitize the eyes of birds (definitive host). Eggs containing miracidia hatch in the water, miracidia penetrate snails (intermediate hosts) and develop into redia and cercariae. When the metacercariae encyst on surfaces of food for birds the infection of a new definitive host can take place by entering the eye or by oral intake [123]. Human cases of philophthalmosis have been reported in Europe (Yugoslavia), Israel, Asia (Thailand, Sri Lanka, Japan) and America (i.e., Mexico, and the United States) [124].

## New or reemerging zoonotic helminths infecting human eyes

Over the last decade, parasitological knowledge has been considerably refined and enhanced by the use of sophisticated technologies and molecular tools, and by the interdisciplinary approach in many fields of the human and veterinary medical sciences. Increasing awareness of physicians on previously poorly known diseases likely is an important part of this process. Reports of several of these infections of the eye have been increasing, but whether this is due to a higher awareness or increasing rates of infection is unclear. Possible reasons for the highest number of reports may include changing epidemiological patterns in the natural definitive hosts, leading to increased exposure of humans, and new geographic range because of spreading into new areas. Three key examples, namely infections by *Angiostrongylus cantonensis*, *Thelazia callipaeda *and *Onchocerca *spp., are discussed below.

### *Angiostrongylus*

Metastrongylids encompass a large group of nematodes (Strongylida, Metastrongyloidae) infecting organs and tissues of different vertebrates [125]. *A. cantonensis*, also known as rat lungworm, is a well recognized zoonotic infection and, as such, is the primary cause of eosinophilic meningitis in Southeast Asia. The infection has spread widely to many other areas of the world, including the Caribbean and Americas [126-130]. The parasite also enters the eye with some frequency. In a review of 484 cases of eosinophilic meningitis, Punyagupta and colleagues [131] noted that 47 (16%) of the cases had reported ocular involvement, and in 7 cases an actively motile worm (most probably *A. cantonensis*) was visualized and removed from the anterior chamber of vitreous of the eye [132,133]. Human ocular infections by larval rat lungworm have been reported in several countries in Southeast Asia [134-143] and, they likely will continue to be reported wherever the parasite occurs, including in new geographical areas such as the Caribbean [144]. Ocular lesions by *A. cantonensis *may either occur alone or may accompany other symptoms such as meningitis [142,145]. Often these long and slender worms reach considerable size in the eye, and are up to a centimeter or more in length [141]. The female worm has a distinctive helical pattern of dark intestine intertwined with light coloured reproductive tubes; male worms have a copulatory bursa and very long (> 1 mm) spicules. These features make it fairly simple to recognize and identify a large worm removed from the eye as *A. cantonensis*.

**Figure 8:**
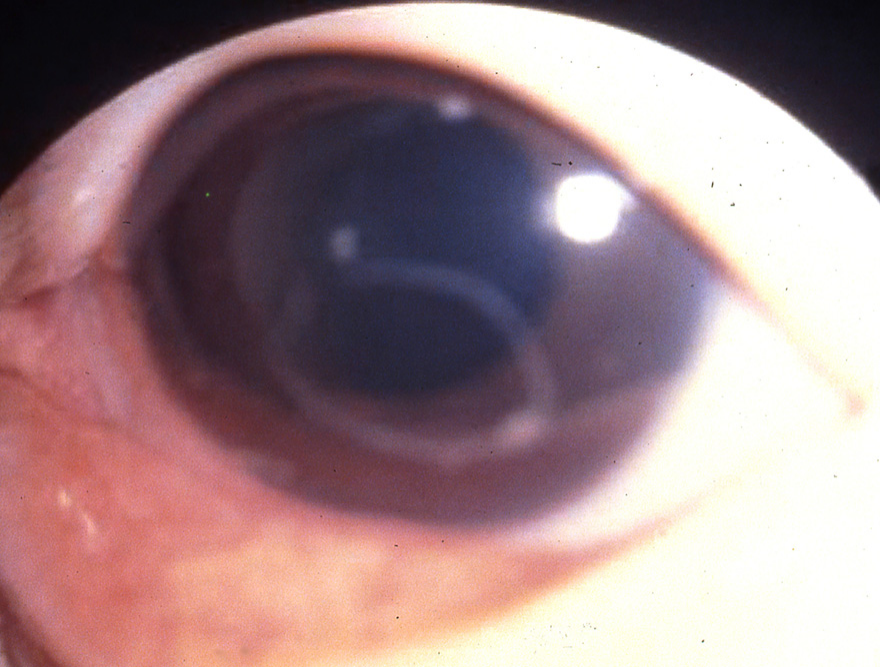
***Angiostrongylus cantonensis *in the anterior chamber of the eye**. *Angiostrongylus cantonensis *in the anterior chamber. (Original by John Cross, courtesy of Lawrence Ash).

Infections are acquired through the ingestion of infected intermediate snail or slug hosts, or a variety of paratenic hosts such as amphibians, reptiles, and some crustaceans that have become infected by ingestion of snails and/or slugs. Migrating larvae in the human host make their way to the CNS, and occasionally into the eye, possibly along the optic nerve. Caution in handling and not eating raw or poorly cooked intermediate or paratenic hosts should prevent most human infections. There is some evidence that people can also be infected by ingestion of produce that has been contaminated with slime trails of snails or slugs into which infective larvae have been shed. Washing produce may help reduce the risk of infection but probably does not remove all risk in endemic areas with high level of transmission. Although in a number of cases, worms are successfully removed, ocular disease caused by larval *A. cantonensis *may vary from blurred vision due to intraretinal haemorrhage [144] to severe optic neuritis due to the presence of the nematode in the vitreous cavity [143]. Other signs include visual disturbances and impairments, extraocular muscular paralysis and a wide range of ocular inflammatory conditions [141,146,147]. Diagnosis is based on the identification of a (usually single) living worm, in any eye localization (e.g., anterior chamber, vitreous cavity, and subretinal space) (Figure 8). The treatment regimen relies on the surgical removal or laser therapy, accompanied by oral benzimidazole (e.g., mebendazole) and corticosteroids in the case of inflammatory manifestatios such as retinitis or optic neuritis [147]. Ocular damage caused by *A. cantonensis *can be severe and may be permanent, thus in some patients the outcome is poor and depends on the initial visual acuity [141,147].

### Thelaziasis

**Figure 9:**
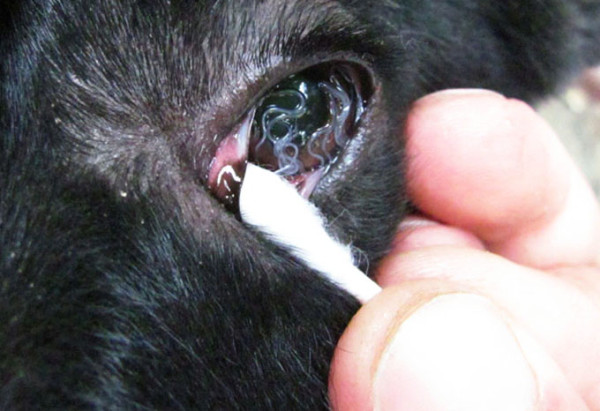
***Thelazia calllipaeda *in a heavily infected dog**. Heavy infection by *Thelazia callipaeda *nematodes in the conjunctiva of a dog from Italy.

**Figure 10:**
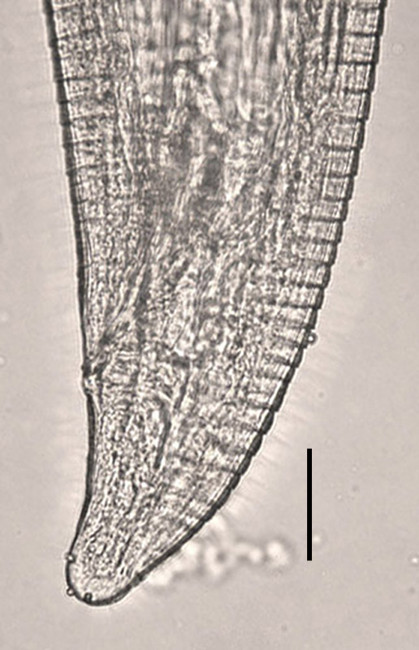
***Thelazia californiensis *from a human patient**. Posterior end of a female *Thelazia californiensis *from the conjunctiva of a human patient in New Hampshire, USA showing cuticle serration. Scale bar = 50 μm. Original; courtesy of DPDx, CDC.

*Thelazia callipaeda *(Spirurida, Thelaziidae) represents a good example of HIE that is both spreading and new to the scientific community in western countries. Along with *Thelazia californiensis *that has been reported to infect humans occasionally in the United States [148], *T. callipaeda *is the only helminth transmitted by secretophagous flies directly into the orbit of humans [149]. This nematode primarily affects the eyes of domestic dogs and cats and wild carnivores (e.g., foxes, wolves, beech martens and wild cats) (See additional file 4: *Thelazia callipaeda *infecting the eye of a dog) [150]. Since its first description at the beginning of the previous century, this nematode has been known as the "oriental eye-worm" for its distribution in the former Soviet Union [151] and the Asian continent, including China [10], Korea [152], Japan [153], Indonesia [154], Thailand [155], Taiwan [156] and India [157]. Human thelaziasis may cause mild to severe clinical signs (including lachrymation, epiphora, conjunctivitis, keratitis and/or even corneal ulcers) [125]. The worm is transmitted by various secretophagous flies which feed on lachrymal secretions of infected animals and/or humans, thus ingesting *Thelazia *1st stage larvae and, after obligate development in the fly, depositing 3rd stage larvae directly back into the orbit. The competence of drosophilid flies of the genus *Phortica *(Diptera, Drosophilidae) as vectors of *T. callipaeda *has recently been elucidated under both laboratory and natural conditions [158,159]. We now recognize that *T. callipaeda *infection is widespread throughout Italy with infection prevalence as high as 60% in dogs from some municipalities (Figure 9) [160] and also in southwestern France (Dordogne area) [161,162] and Switzerland [163]. In addition, four cases of human thelaziasis have been diagnosed in patients coming from an area of north-western Italy and south-eastern France [164]. Infected patients present with exudative conjunctivitis, follicular hypertrophy of the conjunctiva, foreign body sensation, excessive lachrymation, itchiness, congestion, hypersensitivity to light and keratitis, depending on the number of nematodes present in the eye [10]. Children and the elderly seem to be at higher risk. *Thelazia *worms are generally removed intact from the eye, and there are several morphological features that assist in identifying them from other worms that might occur in the orbit, including filaria such as *Loa *or *Dirofilaria*. The morphological identification of *T. callipaeda *has been reviewed [165]. The adult worms measure from 5 to 20 mm in length by 250 - 800 μm in diameter (males are smaller than females). They have a distinct buccal capsule and the cuticle has typical, regularly spaced distinct transverse striations giving the cuticle a ridged appearance (Figure 10). In addition, adult females of *T. callipaeda *are characterized by the position of the vulva located anterior to the oesophagus-intestinal junction, and the males possess five pairs of postcloacal papillae. Poor living conditions and low socio-economic standards seem to be risk factors for acquiring infection, and better hygiene would probably contribute to prevention. The adults and larvae of *T. callipaeda *can be removed mechanically by rinsing the conjunctival sac with sterile physiological saline whereas adults can also be isolated with forceps or cotton swabs [10].

### >*Onchocerca*

**Figure 11:**
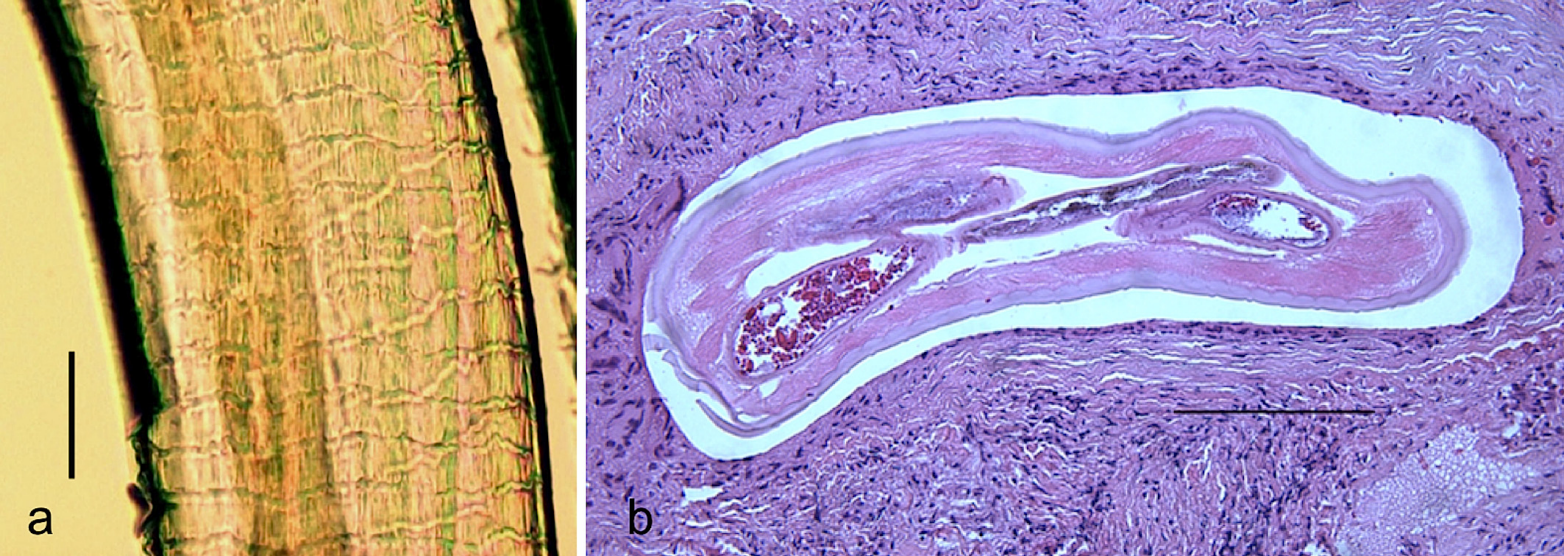
**Zoonotic *Onchocerca *from human ocular connective tissue**. Zoonotic *Onchocerca *sp. from a nodular granuloma of the eye in a patient from Ohio, USA. a) Transverse section of female worm shown in Fig. 5a encased in a nodular granuloma. Low cuticular ridges and inner striae, 2 per ridge, are evident Hematoxylin and eosin stain. Scale bar = 50 μm. (Original; courtesy of Drs. Yassin and Hariri, University of Pittsburg Medical Center). b) Short piece of female *Onchocerca *sp. removed from granuloma tissue before fixation, showing characteristic, diagnostic structures of the cuticle with circular ridges and inner cuticular striae. Scale bar = 150 μm. (Original; courtesy of Drs. Yassin and Hariri, University of Pittsburg Medical Center).

As previously noted, the vast majority of filarioid infections of the eye occur on the conjunctiva, and are caused by species of *Dirofilaria*. However, there is an increasing number of reports of zoonotic *Onchocerca *infections, and several of these have been either within the eye or associated with the conjunctiva or connective tissue of the orbit (See additional file 5: *Onchocerca *sp. infecting the anterior chamber of a human patient). Of the 15 clinical cases reported to date  [166,167], five have been associated with the eye; 3 involved the conjunctiva and 2 involved the cornea. These have been reported from Crimea, the United States, Albania, Hungary, and Turkey [168-172]. The species causing infections of the eye have tentatively been attributed to *Onchocerca gutturosa* or *Onchocerca cervicalis*[169,173], *Onchocerca reticulata *[170], *Onchocerca *spp. [171], and, *Onchocerca lupi *[172]. This last species, *O. lupi*, is of particular interest because it affects dogs and it induces acute or chronic ocular disease characterized by conjunctivitis, photophobia, lacrimation, ocular discharge and exophthalmia [166]. In most cases, zoonotic *Onchocerca *spp. are encased in a nodular granuloma, are resected and sectioned, and the worms identified morphologically (Figure 11a). In section of *Onchocerca *the distinctive muscle anatomy, composed of few, low, poorly developed cells, and the characteristic structures of the cuticle are often apparent, including the circular ridges and inner cuticular striae, making the identification straightforward. The distances between the prominent, undulated annular ridges and the number of transverse striae in the internal layer represent the morphological characters for differentiating filarioids belonging to the *Onchocerca *genus (Figure 11a). The apparent increase in number and range of zoonotic *Onchocerca *infections including those affecting the eye, is noteworthy but difficult to fully explain. Recent cases in both the US and Europe highlight this trend. Case reports of canine ocular onchocerciasis by *O. lupi *[166] have also increased in Europe, including in Greece, Portugal, Germany, Hungary, and Switzerland [174-177]. The number of cases of canine ocular onchocerciasis have also increased in the United States but the species of parasite in the United States has not been established [178-180]. The role played by dogs as reservoir of this zoonotic agent deserves to be investigated further to establish both the primary definitive hosts as well as the vectors that serve to transmit the infection naturally and to humans.

## Diagnosis and cure

The diagnosis of the causative agent is usually only possible after surgery and extraction of the worm or tissue containing the worm, and often requires the assistance of a specialist with an appreciation of the microscopic features of helminths. Generally, in those cases where the parasite is amenable to obliteration with photocoagulation or laser surgery, only a tentative diagnosis is possible. However, surgery remains the only option available for treating a number of the HIE. Although invasive, and often requiring sophisticated devices and advanced medical expertise (not always available in developing countries), ocular surgery is often curative and also allows the recovery of helminths to identify them (Table 3). At the time of ophthalmological examination, observation of motile larvae in the eye is occasionally possible, although a diagnosis at species level can be difficult. Indeed, in some cases it is possible to tentatively identify helminths by measuring larvae in the retina. This is the case with *B. procyonis *larvae which are larger (1 to 2 mm by 50 to 60 μm) than those of *Toxocara *spp. (< 400 by 15 to 20 μm) [47]. This larger size often allows a quick differentiation between *Toxocara *and *Baylisascaris *larvae when seen either in tissue sections or within the eye. Although *Baylisascaris *larvae share several morphologic features in common with *Toxocara*, including single lateral cuticular alae and paired excretory columns, they differ markedly not only in size but in the fact that the gut is patent (has a distinct lumen) in *Baylisascaris *spp. Other types of nematode larvae, such as those of *Gnathostoma *spp., are relatively easy to identify because of their stout, robust size and distinctive head bulb whether they are visualized in the eye or removed. Similarly, *A. cantonensis *worms often reach considerable size in the eye, but are long and slender worms, up to a centimetre or more in length, and are not easily confused with ascarid or *Gnathostoma *larvae, being more likely mistaken for a filarioid. Microscopic examination, however, would quickly allow separation of *Angiostrongylus *from a filaria. Other HIE, such as *Alaria *mesocercaria could be distinguished on the basis of its shape, size (500 × 150 μm), and movement [122]. Their localization on the conjunctiva surface, size, and morphologic features would allow a relatively easy diagnosis of *T. callipaeda*.

**Table 3:** Informative morphological characters and measurements of different stage of helminths infecting human eyes [41,63].

Parasite	Location	Size	Comments	Stage*
*Toxocara canis*	Intraocular	≤ 400 μm × 15-21 μm	Smallest of the nematode larvae encountered in the eye	ML

*Baylisascaris *spp.	Intraocular	1-2 mm × 50-60 μm	Relatively small but easily recognized as being larger than *Toxocara *larvae	ML

*Gnathostoma *spp.	Intraocular	1-5 mm × 200-600 μm	Much more robust than other nematode larva; presence of cuticular spines and head bulb distinctive	ML

*Angiostrongylus *spp.	Intraocular	≤1-2 cm × 200-300 μm	One of the larger, more robust worms found in intraocular location	L, SA

*Thelazia callipaeda*	Eye socket	5-20 mm × 250-800 μm	Distinct morphologic features; free in orbit	A, L

*Philophthalmus*	Intraocular	2-3 mm × 600-800 μm	Ovoid to oblong, flat, solid body	ML, SA

Spargana	Intraocular or eyelid	5-20 mm × 1-2 mm	Long, flat solid body with pseudosegmentation	ML

Coenurus	Intraocular	< 1 cm	Fluid filled cyst ovoid in shape and of variable size	L

*Dirofilaria tenuis/Dirofilaria repens*	Conjunctiva	2-15 cm × 150-400 μm	Males smaller than females; most often closely associated with conjunctiva; worms in the eye are appreciably smaller than those on conjunctiva	L

*Dirofilaria immitis*	Intraocular	1-1.65 cm × 160-400 μm	Not a common location for this worm and very few confirmed cases exist	L
	Conjunctiva	10 cm × 300 μm		SA

*Onchocerca *spp.	Intraocular	3-5 cm × 80-100 μm		L
	Conjunctiva	total length unknown but several cm or more × 150-250 μm		L, SA

*Acanthocheilonema *spp.	Intraocular	1.6 - 2.1 cm × <100 μm	Accurate identification to species has been	L, SA
	Conjunctiva	3.2 cm × <150 μm		

*Larva = L; Migrating larva = ML; SA = Subadult; A = Adult

On the other hand, when HIE are enclosed in a granuloma or within the subconjunctiva, the identification is more difficult. Furthermore, in certain situations, removal may increase the risk to the patient, as in the case of the cystic forms of *Echinococcus *spp. which could induce anaphylactic immunoreactions when disturbed. Indeed, although rare, the localization of *Echinococcus *spp. cysts in the eye are always cause of severe disease, thus the careful surgical removal of the cysts is the only option.

Where appropriate tests exist, serological diagnosis can often contribute to a definitive diagnosis of infection, such as in the case of some ascarids for which serological testing using a sensitive, specific enzyme immunoassay (EIA or ELISA) is available. Serological testing is available for baylisascariasis and can be very helpful in identifying and confirming infection, and, like for toxocariasis, in conducting serosurveys to document the degree of exposure in different populations. Unfortunately, individuals may not mount a measurable immune response during the early phases of acute infection and serologic testing will not provide conclusive evidence to help guide treatment, hence the need for aggressive presumptive treatment in cases with solid exposure history [46]. Serologic assays can be very helpful to confirm infections caused by *Gnathostoma*, especially in cases where no larva or tissue is available to examine. Conversely, seropositivity to spargana in IFAT or ELISA tests always needs to be confirmed by histological examination [104,105].

Unfortunately, surgery is often the only effective treatment for many HIE (e.g., ocular sparganosis, *A. cantonensis*) and this is one of the reasons why these infections represent a traumatic event for the patients and treatment is not a particularly cost-effective manner in which to manage the infection. In some cases, such as the typical zoonotic filarial infection, only a single worm is present and the surgical removal is both therapeutic and curative. In other instances, most notably OLM or larval tapeworms, there is some likelihood that additional larval stages may exist and chemotherapy may be indicated with corticosteroids in the case of inflammatory conditions such as retinitis or optic neuritis [147].

It should be noted that the use of photocoaggulation and laser ablation continue to prove useful in a number of cases infection with HIE, e.g., ascarid larvae, *Alaria *mesocercaria, and often result in improved visual outcome while at the same time destroying the invading helminth *in situ *[122].

## Concerns, ignorance and new avenues linked to HIE

Ophthalmologists and physicians often lack an in- depth knowledge of parasites, rendering it difficult for them to correctly address the etiological identification, treatments and control strategies for many HIE. In addition, the scientific information on HIE available in the international literature is scarce and limited to single case reports in which a clear comparative differentiation among helminth infections is not considered. The main limitation for correctly identifying the etiological agent is that often helminths are not removed, or they are seriously damaged during the surgical procedures thus rendering an accurate morphological identification difficult, if not impossible. The microscopic identification of helminths at the species level often relies on the examination of key morphological characters, not all of which are present on any given specimen or not recognized by the person making the examination, sometimes resulting in an incorrect diagnosis. Accurate identification is crucial to understanding both the source of infection and environmental risks, as well as prescribing correct treatment options.

There are several cases in the literature in which helminths were erroneously identified; for example, cases of *Trichinella *sp. in the vitreous of a woman and *Toxocara *sp. in the retina of a man both from Germany, and a case *Angiostrongylus *sp. recovered from the anterior chamber of a man from Sri Lanka were all incorrectly identified as filaria (reviewed in [63]). Twenty-eight cases of human dirofilariasis from the Old World were erroneously attributed to *D. immitis*, subsequently reviewed and correctly attributed to *D. repens *[181]. Recently, the helminth causing a case of human intraocular infestation in Japan was erroneously identified as *T. callipaeda *although the picture published in the article portrayed a filarioid [182]. In the same article, the authors stated that the life cycle of *T. callipaeda *remains unclear and discussed the possibility of human infection through the skin or by drinking untreated water. This somewhat implausible hypothesis was already dispelled in the late 1990s [183]. The scant attention of medical researchers towards human thelaziasis may also be attributable to the difficulties in its clinical diagnosis and differentiation from allergic conjunctivitis, particularly when small numbers of adult or larval stages are present in affected patients. More recently, the advent of molecular biological techniques has largely supplemented and enhanced knowledge of parasitologists in areas such as systematics (taxonomy and phylogeny), population genetics and molecular identification, diagnosis and control of some HIE [184]. Indeed, the advent of PCR made it possible to study damaged and incomplete specimens, or fragments of specimens encysted in tissues which otherwise would not be morphologically identifiable [185]. The importance of molecular identification and barcoding approach (by the specific PCR-amplification of the* cox*1 and 12S genes) for the rapid identification of specimens has been emphasized, including for either recognized or yet unknown species. Recently, an integrated DNA barcoding of *cox*1 and 12S markers and morphology approaches was shown to be a powerful tool for the taxonomical identification of many filarioid species even if small nematode fragments were available [184]. In addition, the delineation of Molecular Operational Taxonomical Units (MOUTS) was useful to infer potential new species [184].

Basic parasitological research in this field is often fragmentary due to the fact that experimental human infections are rarely done, and the retrieval of helminths from the patients' eyes may be an infrequent occurrence during the ophthalmologic examination. For a number of these helminths, poor experimental models exist, or, if good models exist, the infections generally do not affect the eye in the same way that occurs in aberrant human infections. Thus, scientific knowledge in this field, as well the information on helminth migration patterns is limited, and often has been gained from studies of the same parasites in other animal models. All the above concerns need to be addressed through basic and applied research. For example, many nematode species have not yet been described and even those that are known are often poorly studied such that there is a lack of basic information on the helminth fauna of wild animals (e.g., *O. lupi*). This is particularly true, but not restricted to, regions of the world, such as the Brazilian Amazon forest, where there is wide biodiversity and a large amount of animal and plant species yet to be described [186]. Consequently, species identification of some groups of HIE, such as filarial nematodes, can be difficult if not impossible. Another example of insufficient information is represented by the unknown risk of zoonotic infection, such as other species of *Baylisascaris* (in addition to *B. procyonis*), that may be considered as potential zoonotic agents [46]. For example *Baylisascaris transfuga*, infecting bears worldwide [45,187] has been reported to produce visceral, neural, or OLM syndromes in mice [188-190], gerbils [45,191], and guinea pigs [192]. In addition, cases of fatal neurological diseases have been reported in a colony of Japanese macaques (*Macaca fuscata fuscata*) housed with American black bears in a safari-zoo in Japan [193]. However, the zoonotic role of this parasite for humans has never been demonstrated. Since bears are frequently kept in zoos and game parks and often have high prevalence of the infection in the population (up to 50-100% of bears harbour this parasite) studies on the zoonotic capacity of this parasitic species would be pivotal for a better understanding of the public health risk [194]. Overall, a better understanding of the biology of a number of HIE is crucial for addressing their prevention.

Better awareness among physicians (including ophthalmologists) in the field of parasitology and more active collaboration with parasitologists would be very helpful in proper diagnosis, control and prevention of HIE. This would also allow a better knowledge of the potential risks for being infected by an HIE agent in a given area as well as exposure when travelling in endemic areas. Physicians and ophthalmologists need increased awareness about the existence of a range of zoonotic helminths other than those natural parasites of humans that might be expected to be found in patients' eyes.

Unfortunately, there is a lack of knowledge about many parasites in the local fauna and limited basic research studies are carried out. Monitoring and periodic surveillance for the infections of both domestic and wild animals is important to provide a better understanding of what potential pathogens exist locally, and to prevent the HIE. This is the case with *B. procyonis *which is an emerging infection in raccoons in the southeastern United States, an area traditionally considered to be at low risk [195,196]. An increasing appreciation of onchocerciasis in domestic and wild animals in Europe and the United States is needed to accurately understand what species exist, what the natural definitive host is and, ultimately, what the risks for human infection are. Veterinarians, physicians, and public health officials all share the need to be alert to the possibility of zoonotic infections inside and outside of traditional high-risk areas. Lastly, we need a better understanding of why some parasites migrate to and occasionally enter the eye, especially given that none of these helminths typically resides in or around the eye.

## Conclusions

Despite scientific advances and new methods for treating helminth infections in the human eye, therapies available to patients are somewhat limited and can only be applied in specific cases. This will lead to improvements in the clinical outcome in some cases, but for the foreseeable future, a number of these HIE have complex clinical presentations that still hold potential for serious outcome, including blindness or death, such as in the case of *B. procyonis *infections, where, despite treatment, neurological outcome is dismal in the overwhelming majority of documented cases [46]. However, many cases of these zoonotic helminth infections are preventable by relatively simple measures of improved health and sanitation conditions and awareness on the part of both public and health care providers. Risks for toxocariasis and baylisascariasis could be significantly reduced through better hygiene and reduction of the amount of animal waste in areas where people, especially children, might come in contact with it. For the foodborne zoonoses, such as angiostrongyliasis, gnathostomiasis and others, proper handling and preparation of foods would minimize the risk of infection. For the vector-borne zoonotic infections, control and prevention is likely going to be much harder, as it involves not only the control of the infection in the definitive animal host, but a concerted control of vectors, which is often outside the control of any individual but almost always done at the community or regional level.

## Competing interests

The authors declare that they have no competing interests.

## Authors' contributions

DO and MLE equally contributed in writing the article.

## Supplementary Material

Additional file 1 **Surgical removal of *Dirofilaria repens *from patient's conjunctiva**. This video shows the surgical removal of *Dirofilaria repens *from the patient's conjunctiva, after topical anaesthesia. The palpebral fissure was maintained open by using the blefarostat, the nematode was extracted after incision of the conjunctiva membranes and it was collected. A shortened version of a video in Otranto D, Brianti E, Gaglio G, Dantas-Torres F, Azzaro S, Giannetto S. **Human ocular infestation by *Dirofilaria repens *(Ralliet and Henry, 1911) in a canine dirofilariosis-endemic area**. *Am Jour Trop Med Hyg *2011 (in press). Click here for file

Additional file 2 **Surgical removal of a *Dirofilaria immitis *-like nematode**. This video shows the surgical removal of a *Dirofilaria immitis *-like female nematode from the anterior eye chamber of a patient from Parà, Brasil. Eye was clipped and the cornea incised with a crescent Beaver corneal knife. The nematode was extracted alive with forceps and Fukasacu hook. The patient recovered without complications after the surgery. A shortened version of a video in Otranto D, Diniz DG, Dantas-Torres F, Casiraghi M, de Almeida INF, de Almeida LNF, Nascimento dos Santos J, Penha Furtado A, de Almeida Sobrinho AF, Bain O **Human intraocular filariasis caused by *Dirofilaria *sp., Brazil. ***Emerg Infect Dis *2011 (in press). Click here for file

Additional file 3 **Surgical removal of *Pelecitus *sp. from the iris fibers of a patient**. This video shows the surgical removal of a *Pelecitus *sp. male nematode (approximately 4 mm in length) from the iris fibers of a patient from the Amazon region, Brasil. After peribulbar anesthesia and corneal incision of 2 mm. The nematode was extracted by aspiration and the surgery had no complication. A shortened version of a video in Bain O, Otranto D, Diniz DG, Nascimento dos Santos J, Pinto de Oliveira N, Negrão Frota de Almeida I, Negrão Frota de Almeida R, Negrão Frota de Almeida L, Dantas-Torres F, Frota de Almeida, Sobrinho E: **Human intraocular filariasis caused by *Pelecitus *sp., Brazil**. *Emerg Infect Dis *2011 (in press). Click here for file

Additional file 4 ***Thelazia callipaeda *infecting the eye of a dog in Basilicata region (southern Italy)**. This video shows *Thelazia callipaeda *nematodes floating in the eye of an infected dog in an endemic area of Italy. Conjunctivitis and lacrymation were the main symptoms observed. In the second part, numerous *T. callipaeda *specimens have been collected by an ocular swab. Click here for file

Additional file 5 ***Onchocerca *sp. infecting the anterior eye chamber of a human patient**. This video shows the occurrence of *Onchocerca *sp. in the anterior chamber of a patient from Colorado, United States. The nematode was surgically removed, extracted alive and identified as *Onchocerca*. The patient recovered without complications after the surgery. A video from the case presented in Burr WE, Brown MF, Eberhard ML: **Zoonotic *Onchocerca *(Nematoda: Filarioidea) in the cornea of a Colorado resident**. *Ophthalmology ***105**:1494-1497, 1998. Video courtesy of Dr. W.E. Burr. Click here for file
